# Complete Genome Sequence of Francisella halioticida Type Strain DSM 23729 (FSC1005)

**DOI:** 10.1128/MRA.00541-20

**Published:** 2020-09-10

**Authors:** Ingrid Uneklint, Caroline Öhrman, Linda Karlsson, Mona Byström, Emil Hägglund, Mats Forsman, Andreas Sjödin

**Affiliations:** a Division of CBRN Security and Defence, FOI–Swedish Defence Research Agency, Umeå, Sweden; University of Maryland School of Medicine

## Abstract

Here, we announce the complete genome sequence of the Francisella halioticida type strain DSM 23729 (FSC1005), isolated from a diseased cultured giant abalone in Japan in 2005. The genome is composed of a 2,197,430-bp-long circular chromosome, with a G+C content of 31.2%.

## ANNOUNCEMENT

In recent years, several novel species of the genus *Francisella*, neighbors to the category A biothreat agent Francisella tularensis, have been reported (1). These species are found within a wide range of ecological niches, although they have high sequence identity. This emphasizes the importance of obtaining high-quality genomes for the design of highly specific detection assays (2). Francisella halioticida was isolated in Japan from the hemolymph of a diseased cultured abalone (Haliotis gigantea) in 2005 (3). A live culture of the strain was obtained from the DSMZ German collection of microorganisms. The strain was grown and sequenced in two rounds, first to render Illumina reads and later to render PacBio reads. For the first round, the strain was grown on McLeod agar, and its DNA was extracted using the EZ1 kit (Qiagen, Hilden, Germany) according to the manufacturer’s protocol. A sequencing library was prepared with a Nextera DNA library prep kit and sequenced with the Illumina MiSeq platform, rendering 840,622 paired-end reads using the MiSeq reagent kit v2 (300 cycles). Prior to analysis, the Illumina reads were trimmed with Trimmomatic v0.36 (4) with the settings LEADING:3 TRAILING:3 SLIDINGWINDOW:4:15 MINLEN:36 and were quality checked with fastqc using default settings. For the second round, the strain was grown on Columbia blood agar containing sea salt. DNA was extracted using the Genomic Tip 100/G kit (Qiagen) following the protocol of the manufacturer and was sent to the Uppsala Genome Center for PacBio sequencing. DNA was sheared to 10 kb using the Hydroshear instrument from Digilab and size selected using a BluePippin instrument on the S1 high-pass 6- to 10-kb cassette. The library was constructed using the SMRTbell template prep kit 1.0, and 163,482 reads were produced on a PacBio RS II instrument using PacBio’s 6th generation of polymerase and 4th generation chemistry. HGAP3 (5), using default settings, generated 113,558 filtered subreads with an *N*_50_ value of 7,931 bp, which were assembled with Canu v1.5 setting the genomeSize to 2 Mbp (6). Circlator clean and fixstart (7), with default settings, were used to remove small and completely contained contigs from the assembly, to trim ends, and to set the start position at the *dnaA* gene. Illumina reads were mapped to the merged chromosome ends with BWA-MEM algorithm v0.7.17 (8) with default settings to verify circularity. A BAM file with Illumina reads mapped to the final assembly, using BWA with the same settings as before, was used as input to two rounds of Pilon (9) using default settings. Annotation of the final assembly was performed using the NCBI Prokaryotic Genome Annotation Pipeline (10, 11).

The final assembly of Francisella halioticida consists of one 2,197,430-bp-long circular chromosome, with a G+C content of 31.18%. The genome contains 2,096 protein-coding sequences, 201 pseudogenes, 10 rRNAs, 40 tRNAs, and 4 noncoding RNAs. As shown earlier (3), *F. halioticida* DSM 23729 (FSC1005) does not belong to any previously known *Francisella* clade. This isolate forms a new separate branching clade in the *Francisella* genus. This updated knowledge is essential for improving assays to be used in epidemiological studies of *Francisella* sp. (2).

### Data availability.

The complete genome sequence for Francisella halioticida DSM 23729 (FSC1005) has been deposited in DDBJ/ENA/GenBank under the accession no. CP022132.1, and the reads have been deposited in the SRA under accession no. PRJNA389776.

## References

[1] Sjödin A, Svensson K, Ohrman C, Ahlinder J, Lindgren P, Duodu S, Johansson A, Colquhoun DJ, Larsson P, Forsman M 2012 Genome characterisation of the genus Francisella reveals insight into similar evolutionary paths in pathogens of mammals and fish. BMC Genomics 13:268. doi:10.1186/1471-2164-13-268. 22727144 PMC3485624

[2] Ahlinder J, Öhrman C, Svensson K, Lindgren P, Johansson A, Forsman M, Larsson P, Sjödin A 2012 Increased knowledge of Francisella genus diversity highlights the benefits of optimised DNA-based assays. BMC Microbiol 12:220. doi:10.1186/1471-2180-12-220. 23009728 PMC3575276

[3] Brevik ØJ, Ottem KF, Kamaishi T, Watanabe K, Nylund A 2011 Francisella halioticida sp. nov., a pathogen of farmed giant abalone (Haliotis gigantea) in Japan. J Appl Microbiol 111:1044–1056. doi:10.1111/j.1365-2672.2011.05133.x. 21883728

[4] Bolger AM, Lohse M, Usadel B 2014 Trimmomatic: a flexible trimmer for Illumina sequence data. Bioinformatics 30:2114–2120. doi:10.1093/bioinformatics/btu170. 24695404 PMC4103590

[5] Chin C-S, Alexander DH, Marks P, Klammer AA, Drake J, Heiner C, Clum A, Copeland A, Huddleston J, Eichler EE, Turner SW, Korlach J 2013 Nonhybrid, finished microbial genome assemblies from long-read SMRT sequencing data. Nat Methods 10:563–569. doi:10.1038/nmeth.2474. 23644548

[6] Koren S, Walenz BP, Berlin K, Miller JR, Bergman NH, Phillippy AM 2017 Canu: scalable and accurate long-read assembly via adaptivek-mer weighting and repeat separation. Genome Res 27:722–736. doi:10.1101/gr.215087.116. 28298431 PMC5411767

[7] Hunt M, De Silva N, Otto TD, Parkhill J, Keane JA, Harris SR 2015 Circlator: automated circularization of genome assemblies using long sequencing reads. Genome Biol 16:294. doi:10.1186/s13059-015-0849-0. 26714481 PMC4699355

[8] Li H 2013 Aligning sequence reads, clone sequences and assembly contigs with BWA-MEM. arXiv 1303.3997 [q-bio.GN]. https://arxiv.org/abs/1303.3997.

[9] Walker BJ, Abeel T, Shea T, Priest M, Abouelliel A, Sakthikumar S, Cuomo CA, Zeng Q, Wortman J, Young SK, Earl AM 2014 Pilon: an integrated tool for comprehensive microbial variant detection and genome assembly improvement. PLoS One 9:e112963. doi:10.1371/journal.pone.0112963. 25409509 PMC4237348

[10] Orvis J, Crabtree J, Galens K, Gussman A, Inman JM, Lee E, Nampally S, Riley D, Sundaram JP, Felix V, Whitty B, Mahurkar A, Wortman J, White O, Angiuoli SV 2010 Ergatis: a Web interface and scalable software system for bioinformatics workflows. Bioinformatics 26:1488–1492. doi:10.1093/bioinformatics/btq167. 20413634 PMC2881353

[11] Haft DH, DiCuccio M, Badretdin A, Brover V, Chetvernin V, O'Neill K, Li W, Chitsaz F, Derbyshire MK, Gonzales NR, Gwadz M, Lu F, Marchler GH, Song JS, Thanki N, Yamashita RA, Zheng C, Thibaud-Nissen F, Geer LY, Marchler-Bauer A, Pruitt KD 2018 RefSeq: an update on prokaryotic genome annotation and curation. Nucleic Acids Res 46:D851–D860. doi:10.1093/nar/gkx1068. 29112715 PMC5753331

